# Scaling Effects on Chlorophyll Content Estimations with RGB Camera Mounted on a UAV Platform Using Machine-Learning Methods

**DOI:** 10.3390/s20185130

**Published:** 2020-09-09

**Authors:** Yahui Guo, Guodong Yin, Hongyong Sun, Hanxi Wang, Shouzhi Chen, J. Senthilnath, Jingzhe Wang, Yongshuo Fu

**Affiliations:** 1 Beijing Key Laboratory of Urban Hydrological Cycle and Sponge City Technology, College of Water Sciences, Beijing Normal University, Beijing 100875, China; guoyh@lreis.ac.cn (Y.G.); guodong_yin@bnu.edu.cn (G.Y.); 201921470001@mail.bnu.edu.cn (S.C.); 2 The Center for Agricultural Resources Research, Institute of Genetics and Developmental Biology, The Chinese Academy of Sciences, 286 Huaizhong Road, Shijiazhuang 050021, China; hysun@sjziam.ac.cn; 3 State Environmental Protection Key Laboratory of Wetland Ecology and Vegetation Restoration/School of Environment, Northeast Normal University, Jingyue Street 2555, Changchun 130017, China; wanghx197@nenu.edu.cn; 4 Institute for Infocomm Research, Agency for Science, Technology and Research (A*STAR), Singapore 138632, Singapore; jsenthilnath@alum.iisc.ac.in; 5 MNR Key Laboratory for Geo-Environmental Monitoring of Great Bay Area of the Ministry of Natural Resources & Guangdong Key Laboratory of Urban Informatics & Shenzhen Key Laboratory of Spatial Smart Sensing and Services, Shenzhen University, Shenzhen 518060, China; Jingzhewang@szu.edu.cn

**Keywords:** scale effects, maize, UAV/UAS, SPAD, chlorophyll contents, HSV, machine learning

## Abstract

Timely monitoring and precise estimation of the leaf chlorophyll contents of maize are crucial for agricultural practices. The scale effects are very important as the calculated vegetation index (VI) were crucial for the quantitative remote sensing. In this study, the scale effects were investigated by analyzing the linear relationships between VI calculated from red–green–blue (RGB) images from unmanned aerial vehicles (UAV) and ground leaf chlorophyll contents of maize measured using SPAD-502. The scale impacts were assessed by applying different flight altitudes and the highest coefficient of determination (R^2^) can reach 0.85. We found that the VI from images acquired from flight altitude of 50 m was better to estimate the leaf chlorophyll contents using the DJI UAV platform with this specific camera (5472 × 3648 pixels). Moreover, three machine-learning (ML) methods including backpropagation neural network (BP), support vector machine (SVM), and random forest (RF) were applied for the grid-based chlorophyll content estimation based on the common VI. The average values of the root mean square error (RMSE) of chlorophyll content estimations using ML methods were 3.85, 3.11, and 2.90 for BP, SVM, and RF, respectively. Similarly, the mean absolute error (MAE) were 2.947, 2.460, and 2.389, for BP, SVM, and RF, respectively. Thus, the ML methods had relative high precision in chlorophyll content estimations using VI; in particular, the RF performed better than BP and SVM. Our findings suggest that the integrated ML methods with RGB images of this camera acquired at a flight altitude of 50 m (spatial resolution 0.018 m) can be perfectly applied for estimations of leaf chlorophyll content in agriculture.

## 1. Introduction

Maize (*Zea mays* L.) is a global stable crop that accounts for more than 34% of global cereal production, and the demand of it is constantly increasing with the growth of the global population and the impending economic pressures in the coming decades [1,2,3]. China has contributed 17% of global maize production with less than 9% of arable cropland considering the environmental and ecological protection [4,5,6,7]. Climate change, such as increasing temperature and abnormal precipitation, has both directly and indirectly influenced the growth and development of maize, which will inevitably result in the reduction or the stagnation of yields [8,9,10,11]. Thus, timely monitoring of the growth condition of maize and making adaptive measures are essential for guaranteeing the agricultural production and ensuring national food security. Chlorophyll is the most important pigment in plant photosynthesis that can reflect the strength of crop photosynthesis, the quality of nutrition, and physiology [12,13,14]. Therefore, chlorophyll content can be used for assessing, monitoring, and evaluating the growth status of crops. The precise measurement of chlorophyll content using SPAD-502 is very high, which is almost the same as the result using chemical tests, thus the chlorophyll contents measured by SPAD-502 can be perfectly used for replacing the chlorophyll in vegetation [15,16]. Thus, calculating and estimating the chlorophyll contents at the field scale are prerequisites for monitoring crop growth and strengthening decision-support systems for specific agronomic practices (e.g., fertilization, irrigation, weeding, ploughing, and harvest) [17,18,19].

Commonly, there are three approaches for measuring the chlorophyll contents of vegetation at field scale: destructive sampling (DS), simulation models (SM), and remote sensing (RS). The DS method is a direct method that is quite precise in measuring the chlorophyll contents of crops using experiments, but it is also very labor-intensive, time-consuming, and inefficient. Thus, it can hardly be applied for a relatively large area and the sampling points are also limited. Moreover, it is very destructive and time-consuming to acquire the data for a large area. The SM method is a natural laboratory that simulates the whole growing process of crops covering the status of all variables of crops such as chlorophyll content, but this method relies on the high resolution of input data such as weather, soil, management practice that are difficult to obtain [20,21,22]. Alternatively, RS has been successfully applied in many related fields such as image classification and change detection. In addition, the advanced RS techniques such as unmanned aerial vehicle remote sensing (UAV-RS) can be applied to acquire filed observation data at a fine spatial resolution (centimeter-level). Unlike the traditional satellite remote sensing (SRS) that is commonly limited by the spatial and spectral resolutions, and long revisit cycle, the UAV-RS can provide images at adequate spatial and temporal resolutions without the limitations from the weather condition [23,24,25]. It is also important for agricultural and ecological applications as they can be easily deployed and possessed the ability to dynamically monitor the crops in detail during important phenology events such as flowering, heading, and mature that are the critical growth stages of crops. The remote sensed sensors mounted on the UAV could fill the gap between high resolution in spatial and the quick revisit circle [26]. Thus, UAV-RS combined field data collection is the best choice that can acquire the database of complete growth of crops at high spatial and temporal resolution with less time.

In the red–green–blue (RGB) color space system, each pixel is defined using the combined R, G, and B band [27]. The vegetation index (VI) calculated from RGB images have been used to monitor the leaf chlorophyll content of crops for several decades [28,29,30,31]. Thus, cost-effective RGB cameras onboard UAVs have great abilities in monitoring the growth conditions using visual vegetation index for agricultural and ecological applications [32,33]. Rocio Ballesteros et al. monitored the biomass of onion using RGB images acquired from UAV platform [34]. Alessandro Matese et al. assessed the intra-vineyard variability in terms of characterization of the state of vines vigor using high spatial resolution RGB images [35]. Dong-Wook Kim et al. modeled and tested the growth status of Chinese Cabbage with UAV-Based RGB images [36]. The RGB and multispectral images from UAV were used together for the detection of the Gramineae weed in rice fields [37]. Jnaneshwar et al. clearly described the workflow of monitoring the plant health, crop stress and the guidance of management using multispectral 3D imaging system mounted on a UAV [38]. Zarco-Tejada et al. combined the helicopter-based UAV with the multispectral imaging sensors six-band multispectral camera (MCA-6, Tetracam, Inc., Chatsworth, CA, USA), and the images acquired with this system were first calibrated using linear regression method and further applied for the extraction of a series of vegetation index for agricultural parameter estimations [39]. Jacopo et al. performed the flight mission at a site-specific vineyard with Tetracam ADC-lite camera (Tetracam, Inc., Gainesville, FL, USA), and the ground measurement using FieldSpec Pro spectroradiometer (ASD Inc., Boulder, CO, USA) was applied for radiometric calibration [40]. Miao et al. assessed the potential ability of hyperspectral remote sensing images acquired with an AISA-Eagle VNIR hyperspectral imaging sensor (SPECIM, Spectral Imaging, Ltd., Oulu, Finland) through building multiple regression analyses between the bands from images and measured values using SPAD-502, and the results showed that bands from images explained 68–93% and 84–95% at the fields in the corn-soybean and corn-corn rotation fields, respectively [41]. Wang et al. estimated the leaf biochemical parameters in mangrove forest using hyperspectral data [42]. The multispectral images from multispectral and hyperspectral cameras had advantages in agricultural applications; however the cameras were relatively expensive compared with RGB cameras [43]. To date, only a few studies assessed and evaluated the chlorophyll contents using RGB images acquired from the UAV platform, and thus the ability and performance of RGB in predicting chlorophyll contents are still unexplored. The information extracted from high resolution images is enough for information mining, and there is no guarantee that the extracted information from all pixels is reasonable and true. The images acquired from different altitudes representing the different image resolutions. Thus, the resolution should match the ground samples and the flight altitude influencing the resolution of images from should be optimized to better achieve the data fitting [44]. The scale impacts using RGB images acquired from different imaging environment such as imaging at different flight altitudes were little evaluated. Meanwhile, the RGB cameras were relatively cheaper and more easily to be deployed than the multispectral cameras. Also, the previous adopted statistics approaches were traditional linear regression models, where there is a lack of learning underlying data distribution [15]. The statistical regression models using traditional linear regression models are mostly localized, and this issue can be overcome using more advanced machine-learning (ML) techniques such as backpropagation neural network model (BP), support vector machine (SVM), and random forest (RF). ML has been successfully applied in many domains including image pre-processing, image classification, pattern recognition, yield prediction, and simulation regression. The BP, SVM, and RF have been applied for many applications as they perform better for regression problems, especially the SVM method have been reported to have achieved the highest precision in previous studies [45,46].

In this study, we are trying to address the following: (1) investigating the scale effects using UAV RGB images acquired from different flight altitudes at the early growth stage of maize; (2) evaluating the performance of hue–saturation–value (HSV) color system compared with RGB color system in applications such as information extraction using vegetation index; (3) estimating the chlorophyll contents using ML methods with RGB images from different growth stages of maize.

## 2. Materials and Methods

### 2.1. Study Area

**Figure 1:**
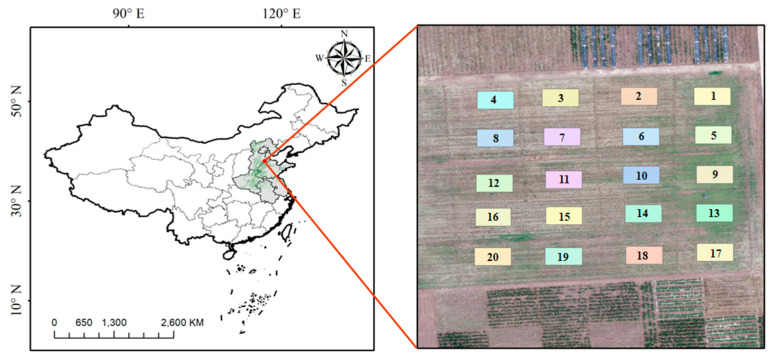
Nanpi Eco-Agricultural Experimental Station (NEES) and overview of the long-term experimental fields.

The experiment of different treatments of fertilizers to maize was conducted in Nanpi Eco-Agricultural Experimental Station (NEES) (38.00°N, 116.40°E), which was managed by the Chinese Academy of Sciences (CAS) (Figure 1). The NEES was in Hebei Province belonging to the North China Plain (NCP), which was the national main grain product areas of summer maize and winter wheat. The general growth duration of maize was from middle June to early October in a single year. There was a total of 20 plots in this area, and each plot was treated using different amount of fertilizers containing the common usage of nitrogenous fertilizer, phosphate fertilizer, and potassium fertilizer, respectively (Table A1). The study area was in the semi-humid monsoon climate zone, and the annual average temperature and average annual precipitation is 12.3 °C and 480 mm, respectively. The soil type of this region belonged to the cinnamon soil subgroup. The parent material of this soil species was deep and uniform, of which the profile is A-AB-BK. The A layer (0–20 cm) was desalinated Chao soil, with some saline soil. The AB layer (20–48 cm) was sandy coarse loam with a granular structure and the BK layer (48–100) was clay loam with weak adhesion. The soil in NEES represented the typical type of water-salt salinization in this region. 

### 2.2. Data Collection and Pre-Processing

#### 2.2.1. UAV Data Collection and Pre-Processing

The UAV flights covering all 20 plots in NEES were carried out between 11:00 and 11:30 AM on 8 July, 18 August, 1 September, 16 September 2019, respectively. The flight on July 8 contained five different altitudes: 25 m, 50 m, 75 m, 100 m and 125 m. The flight altitudes on 18 August, 1 September, 16 September were all set as 50 m. The DJI Phantom 4 Pro V2.0 was used as the UAV platform for data collection, of which the max ascent speed and max decent speed were 6 and 4 m per second, respectively. The horizontal and vertical accuracy ranges were ±0.1 m and ±0.3 m (with vision positioning) with the electronic shutter speed as 1/8000 s. The platform had an endurance of up to 30 min, and the camera data storage capacity was approximately six hours. The RGB camera had a focal lens of 8.8 mm and a 20.7 megapixel (5472 × 3648 pixels) CMOS sensor arranged through the same lens. Before the flight missions, four ground control point (GCP) were made as prominent positions on the ground using white paints, and the precise locations were measured using Real-time kinematic (RTK) S86T system. To acquire the RGB images from different flight altitudes, the commercial software Altizure (V4.7.0.196) was applied for flight control with 85% forward lap and 75% side lap for all flight mission. Each flight mission had covered the whole experimental field, with the four GCPs included. Since the light conditions were very crucial for image capture and image processing in remote sensing domains for quantitative remote sensing. The sunny days were selected for data collection, and thus the impacts of solar and other disturbance of cloud was minimized. To assess the scale impacts, the data was collected in one single day within one hour to exclude the impacts of different solar radiation and angles. In addition, to better assess the scale impacts ascribed from different flight altitudes, an implement experiment was added on 16 July 2020 and the same approach of different flight altitudes were conducted the same as on 8 July 2019. The experiments with the acquisition of UAV images were also conducted on 18 August, 1 September, 16 September 2019 with the same method, and the only difference was that the flight altitude was set as 50 m. The RGB images acquired from different flight altitudes and on different dates were copied and transferred from the storage card mounted on the UAV. The standard procedure was conducted within Pix4d mapper using GCPs, which was a unique photogrammetry software suite for drone mapping [47,48,49].

#### 2.2.2. Chlorophyll Field Measurements Data

The ground collection of chlorophyll contents in each plot was conducted using SPAD-502 under a standard procedure. The relative amount of leaf chlorophyll content was determined by measuring the light transmittance coefficient of the leaf at two wavelengths: 650 nm and 940 nm. The values measured using SPAD-502 was closely correlated with the chlorophyll content in leaf of plants, and the trend of chlorophyll content can be known by measured values. To eliminate the errors and make the measures more reliable, five points measuring method containing four corners and the center of each plot were measured for three repetitions. The average of chlorophyll contents of each plot can be precisely acquired by averaging the 15 (5 × 3 = 15) samples of data. Since there was a total of 20 plots, there were 20 values of chlorophyll contents after the average calculation of each flight. The chlorophyll contents measured by SPAD-502 were carried out on 8 July, 18 August, 1 September, 16 September shortly after the flight missions.

### 2.3. Methods

#### 2.3.1. Scale Effects Using Vegetation Index Methods

The mosaic image acquired at different altitudes on July 82019 were shown in ENVI 5.3 and the spatial resolution were calculated. The spatial resolutions for flight altitudes of 25, 50, 75, 100 and 125 m were 0.006, 0.018, 0.021, 0.028, 0.034 m, respectively. The mosaic image of 50 m was shown in ENVI 5.3 and the region of interests (ROI) covering the center and four corners within each plot was made and exported, respectively (Figure 1). The same ROI of each plot were used to extract the subsample images within each plot of mosaic images including the long time series (8 July, 18 August, 1 September, 16 September) and different flight altitudes (25, 50, 75, 100, 125 m), respectively. Thus, there are 20 subsample images for each flight altitude and each date. A total of 18 indices had been applied and conducted in previous studies (Table 1). Before calculating the indices, the R, G and B bands were normalized using,

R = r/(r + g + b), G = g/(r + g + b), B = b/(r + g + b)(1)

where r, g, and b represented the original digital number (DN) of the RGB images. Thus, the R, G and B represented the normalized DN that can be used for calculating vegetation index and quantitative remote sensing analyses.

The HSV color system converted the red, green, and blue of RGB images into HSV, of which the hue represents the value from 0 to 1 that corresponds to the color’s position on a color wheel. As hue increased from 0 to 1, the color transitions from red to orange, yellow, green, cyan, blue, magenta, and finally back to red. Saturation represented the amount of hue or departure from neutral. In addition, 0 indicated a neutral shade, whereas 1 indicated the maximum of saturation. The HSV value represented the maximum value among the RGB components of a specific color.

The scale impacts were mainly due to the different resolution of images, and in this study, we only focused on the scale impacts of different resolution ascribed from different flight altitudes. To assess the scale impacts, vegetation index in Table 1 were calculated using the RGB images acquired from different flight altitudes. Three approaches were conducted and compared, with the consideration of elimination of the background effects such as disturbance from soil and the color space system. For the first approach, the 20 plots of subsample images were used directly to build linear regression models with the measured chlorophyll contents using SPAD-502 at each plot, and images from different flight altitudes and different dates were separately assessed using the regression function in Matlab 2019b. The R^2^ was obtained for each vegetation index and compared with the results from different flight altitudes. The results using the first approach showed much irregularity that was mainly due to the impacts of background such as soil and other occlusions. For the second approach, the EXG-EXR method was applied to extract only green pixels and to reduce the effects of background disturbance such as soil [52,82]. In this way, the subsample images were classified into green pixels and non-green pixels. The subsample images were then transformed into binary images where the DN of green were assigned as 1 and the DN of background were assigned as 0. Thus, the pixel values equal 1 corresponding to green were used to build linear regression models with the measured chlorophyll contents in each plot. For the third approach, the subsample images were first classified into green and non-green pixels using the second approach, and the classified images (only green pixels) in RGB color space system were transformed into HSV color space system, which was an alternative representation of the RGB color space [83,84]. The HSV model was invented to align with the way human vision perceives color-making attributes, and the colors of each hue were arranged in a radial slice, around a central axis of neutral colors which ranged from black at the bottom to white at the top [85,86]. The subsample images in RGB color space were converted into HSV color space, and the binary images were used for extraction of VI using the green pixels. Thus, the images in HSV color space without the effects of background were used to build linear regression models with the measured chlorophyll contents at each plot using images from five flight altitudes. These three approaches were used to systematically evaluate and investigate the effects of background such as soil and to assess the performance of RGB and HSV color space.

#### 2.3.2. Estimating the Chlorophyll Contents Using Machine-Learning Techniques

To precisely predict the chlorophyll contents, the advanced ML methods: BP, SVM and RF were used to build non-linear relationships. The independent variables were the 18 vegetation indices and the dependent variable was the chlorophyll content in each plot. For all ML models, 70% of samples were selected for building models and the remaining 30% of samples were used for validations. Moreover, the ten-fold cross-validation was adopted to assess model validity. The results using different ML models were obtained and compared with each other. Furthermore, all samples were adopted to build non-linear relationships to predict the chlorophyll contents of each pixel using all subsample images, and the chlorophyll contents at the site scale can be precisely acquired through this way. To assess the model performance and evaluate the prediction accuracy, the coefficient of determination (R^2^), root mean square error (*RMSE*), and mean absolute error (*MAE*) between observed and simulated yield of maize was applied. The equations are defined as follows:

(2) $$R^{2}=\frac{\sum_{i=1}^{n}\left(M_{i}-\bar{M}\right)*\left(P_{i}-\bar{P}\right)}{\sqrt{\sum_{1}^{n}{\left(M_{i}-\bar{M}\right)}^{2}*\sum_{1}^{n}{\left(P_{i}-\bar{P}\right)}^{2}}}$$

(3) $$\;\;RMSE=\sqrt{\frac{1}{n}\sum_{1}^{n}{\left(P_{i}-M_{i}\right)}^{2}}$$

(4) $$MAE=\frac{\sum_{i=1}^{n}\left|P_{i}-M_{i}\right|}{n}$$

In the equations, R^2^ is the coefficient of determination, *n* represents the total number of samples, *M_i_* represents the true values, and *P_i_* represents the predicted values. $\bar{M}$ and $\bar{P}$ represent the average of *M* and *P*, respectively.

## 3. Results

### 3.1. The Results of Scale Impacts Using Images from Different Flight Altitudes

**Figure 2:**
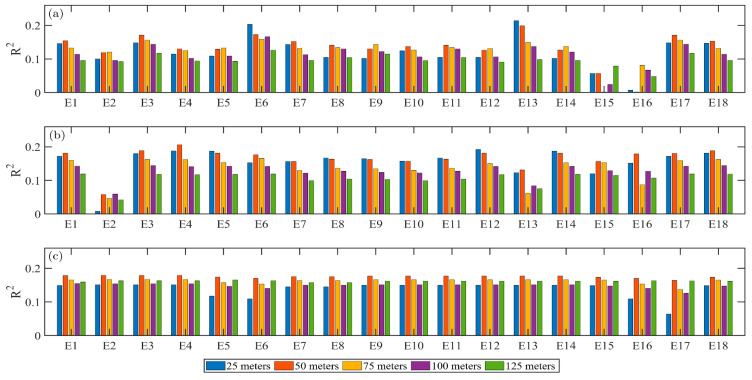
Histogram of coefficient of determination (R^2^) between index and chlorophyll contents using E1–18 (Table 1) using, (**a**) RGB color space with background; (**b**) RGB color space with only green pixels; (**c**) HSV color space with only green pixels.

**Figure A1:**
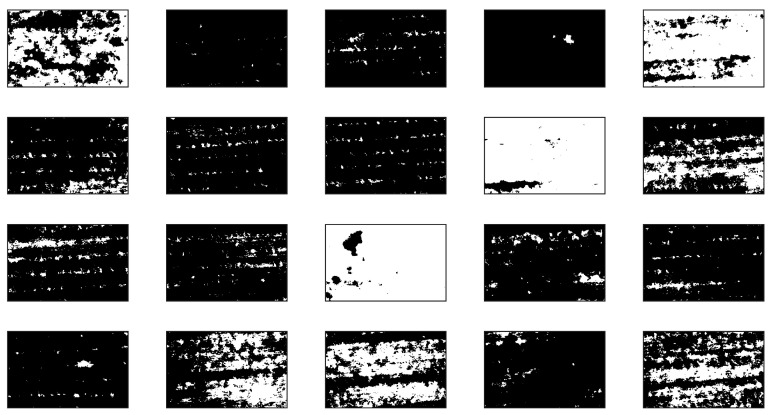
Crop binary map at flight altitude at 50 m using EXR-EXG method.

The 18 VI were used to build linear relationships with chlorophyll contents in each plot for different flight altitudes (Table A2, Table A3, Table A4). For the data acquired on July 8 in 2019, the crop binary map at flight altitude 50 m using EXR-EXG method is shown in Figure A1. The results of R^2^ between VI and SPAD values using images acquired from different flight altitudes on 8 July 2019 were each obtained and compared (Figure 2). It can be concluded that the R^2^ calculated using E 6 and E 13 performed better (Figure 2a). The percentage of VI with corresponding of the R^2^ increased from flight altitude of 25 to 50 m and gradually decreased from flight altitude of 50 to 125 m were 44% and 50%, respectively (Figure 2a,b). Thus, the proposed second approach was better than the first approach, and the significantly increased R^2^ had indicated that the elimination of effects of background had improved the accuracy to some extent. The results in Figure 2b had removed the disturbance of background such as soil. When the HSV color space was applied, the percentage of VI of R^2^ increased from 25 to 50 m has increased to 100%, which implied that the images in HSV color space system were better for information extraction than the traditional RGB color space system, especially for estimating the chlorophyll contents.

**Figure 3:**
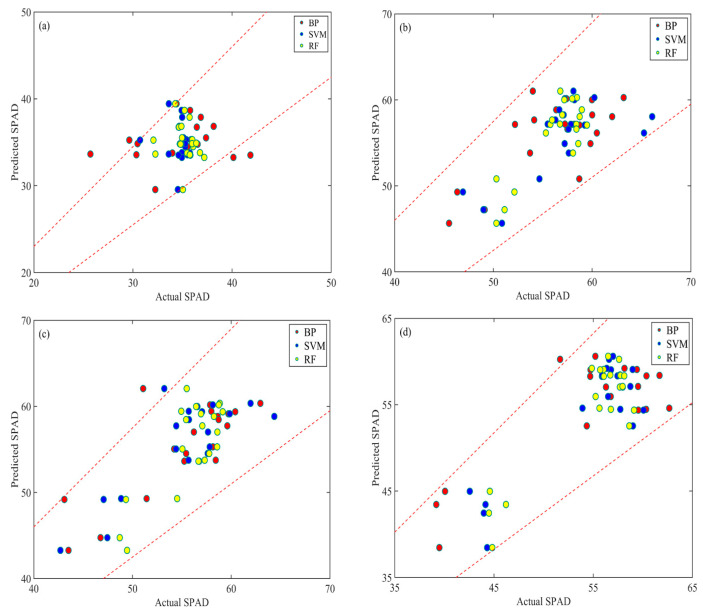
Chlorophyll contents predictions using ML methods. The red, blue, and yellow points are the predicted values of the chlorophyll contents using BP, SVM, and RF, respectively. (**a**–**d**) represents the results using images acquired on 8 July, 18 August, 1 September, 16 September 2019.

**Figure A2:**
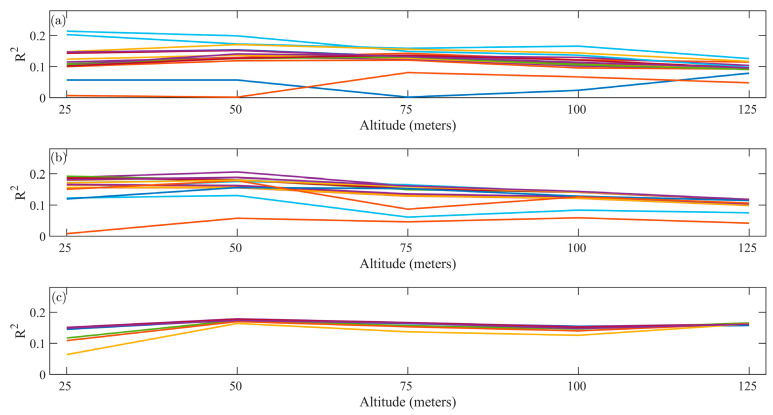
Polyline of R^2^ between index and chlorophyll contents using E1-E18. (**a**) RGB color space system with background; (**b**) RGB color space system without background; (**c**) HSV color space system without background.

The results shown in polyline forms of five flight altitudes also indicated that the proposed second approach was better than the first approach (Figure A2). Even though some of the R^2^ using the first approach were larger than that of the second approach, the background such as soil had covered the real phenomenon as the images were acquired at the early growth of maize. The increased percentage of R^2^ using the second approach was quite evident. Also, the results in Figure 3c clearly demonstrated that the HSV was much better than the RGB. The percentage of R^2^ increased significantly from 20 to 50 m and then decreased when the HSV color space was applied for regression. Thus, the spatial resolution of images acquired at flight altitude 50 m was better matched with the ground collection resolution which were least affected by the scale impacts. The HSV color space system may have greater potential in the analysis of quantitative remote sensing than the common RGB color space system. 

**Figure A3:**
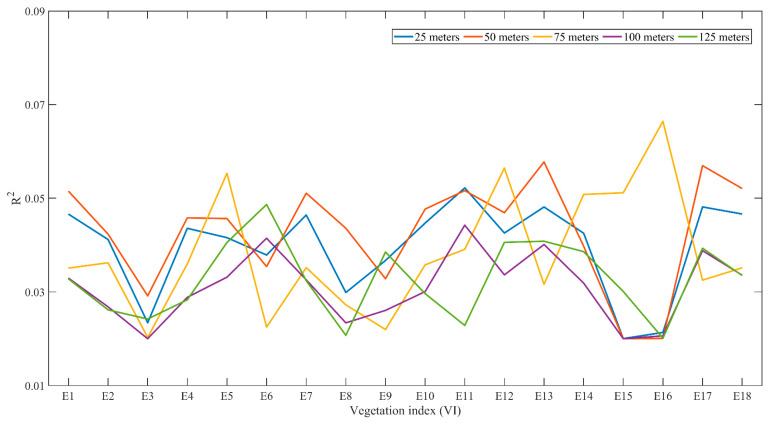
The linear regression results between vegetation index and measured values using SPAD-502.

For the experiment conducted on 16 July 2020. The VI were all used to build linear regression relationships with the values measured by SPAD-502. Again, the results showed that the images acquired from flight altitude of 50 m were the least influenced by the scale impacts, where the R^2^ from 50 m were the highest compared with the R^2^ from other flight altitudes, respectively (Figure A3). The average values of R^2^ were 0.040, 0.043, 0.038, 0.031, 0.033 for 25, 50, 75, 100, 125 m, respectively. Thus, the images acquired from 50 m were least influenced by the scale impacts from different flight altitudes.

### 3.2. Performance of Machine-Learning Methods and Chlorophyll Contents Prediction

Since the images acquired from flight altitude of 50 m were least impacted by the scale impacts, thus the following section was performed using the images acquired at flight altitude of 50 m. The R^2^ between chlorophyll contents and VI calculated using RGB images acquired on 8 July, 18 August, 1 September, 16 September 2019 were obtained (Table 2). The dates of 8 July, 18 August, 1 September, 16 September 2019 represented different growth stages of maize, and the R^2^ between the VI and chlorophyll contents increased significantly with the growth of maize. The highest value of R^2^ was the linear relationship between chlorophyll contents and E5, which had reached 0.845. Thus, the VI extracted from RGB images had great potential for chlorophyll content estimations.

The RGB images acquired on 8 July, 18 August, 1 September, 16 September 2019 were used for extraction of subsample images using the ROIs of each plot. Then the VI were obtained for each plot, and the ML methods were applied between the VI and chlorophyll contents from ground measurement. The models were all trained using 70% of samples and validated using the remaining 30% samples. The predicted values and actual values of chlorophyll contents were obtained with the ±15% error lines, and most of the points were within the error lines indicated the predictions using ML methods were relatively high (Figure 3). The scatter points those were out of the error lines were all predicted values using BP, which indicated the SVM and RF performed better than the BP. 

The detailed assessments of results between actual and predicted chlorophyll contents including R^2^, RMSE and MAE for each model and each date are shown in Table 3. The average of R^2^, RMSE, and MAE were 0.001, 2.996 and 2.316 for 8 July, 0.337, 3.216 and 2.553 for 18 August, 0.549, 3.357 and 2.642 for 1 September, 0.668, 3.579 and 2.882 for 16 September, respectively. The R^2^ increased with the increase of days, and the highest values can be obtained on 16 September. The calculated RMSE and MAE of all models and all dates were less than 5, thus the ML models were efficient for chlorophyll contents predictions.

**Figure 4:**
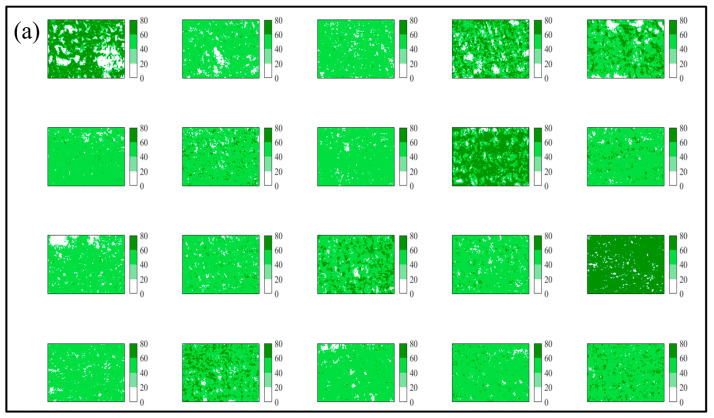

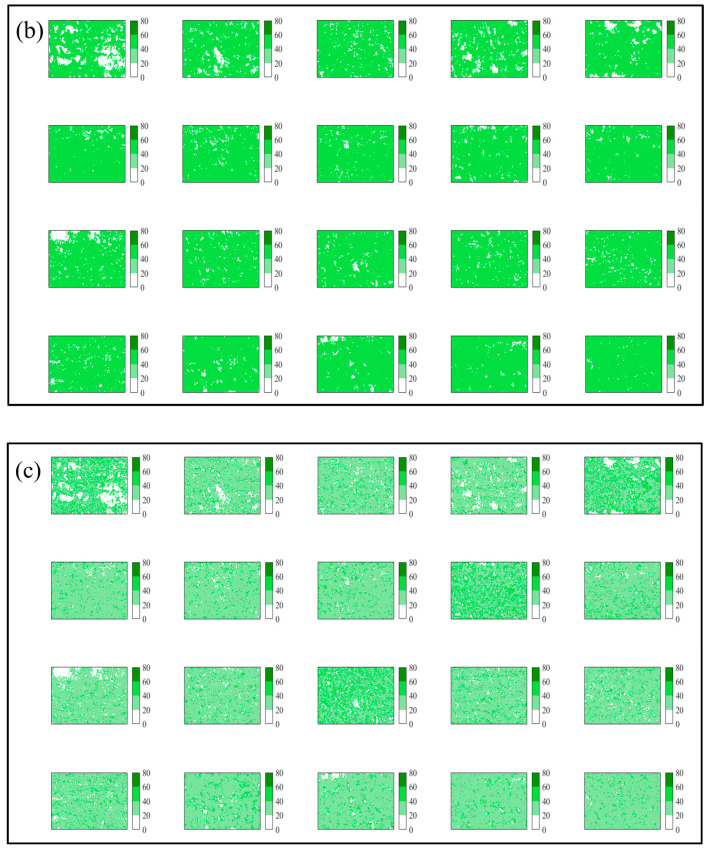
The predicted chlorophyll contents values by BP, SVM, and RF models using images acquired on 16 September 2019. Note: (**a**–**c**) represented the predicted chlorophyll contents using BP, SVM and RF, respectively.

**Figure A4:**
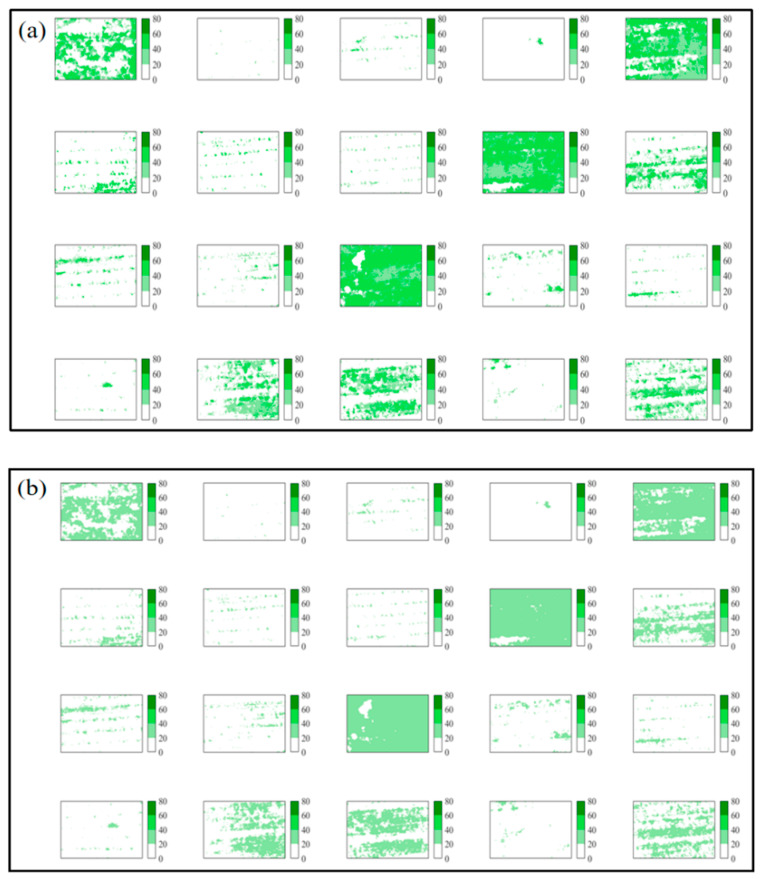

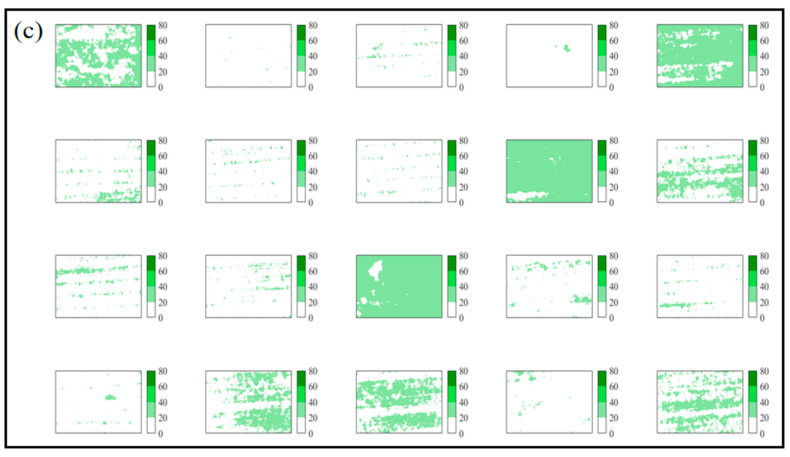
The predicted chlorophyll contents values using BP, SVM, and RF models using images acquired on 8 July 2019. (**a**–**c**) represented the predicted chlorophyll contents using BP, SVM, and RF, respectively.

**Figure A5:**
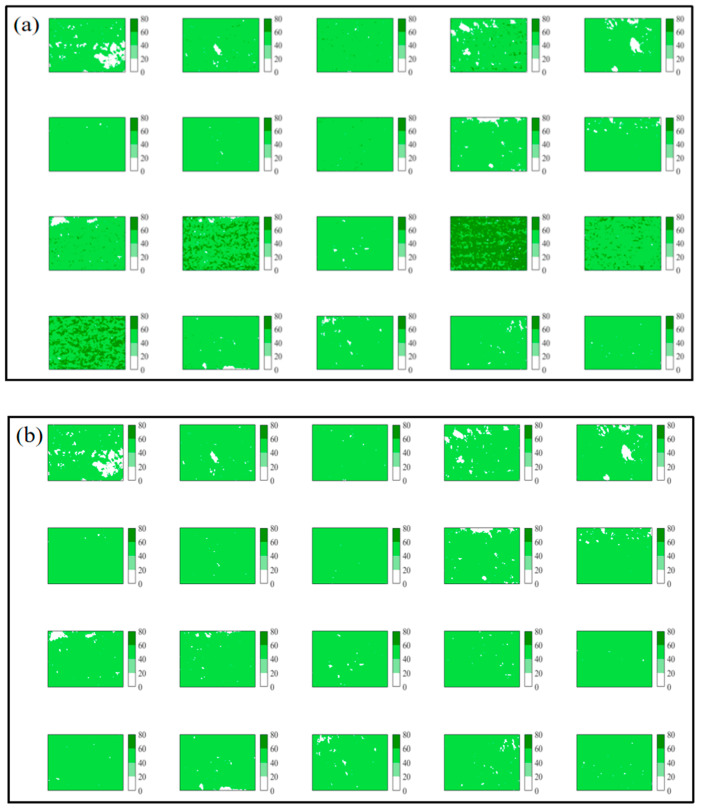

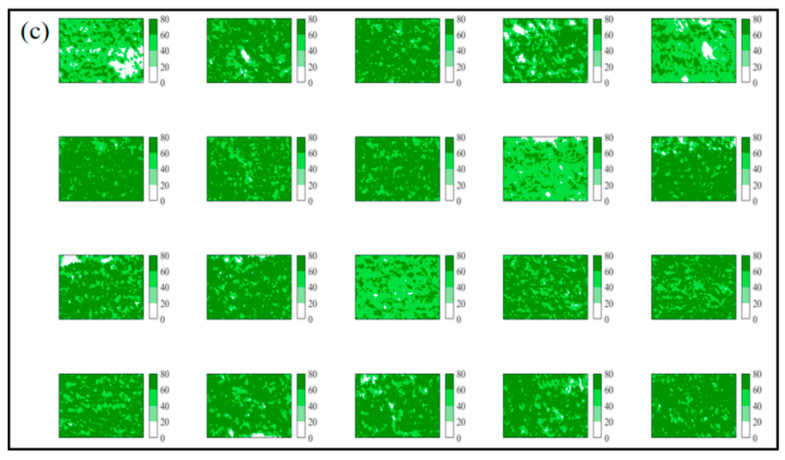
The predicted chlorophyll contents values using BP, SVM, and RF models using images acquired on 18 August 2019. (**a**–**c**) represented the predicted chlorophyll contents using BP, SVM, and RF, respectively.

**Figure A6:**
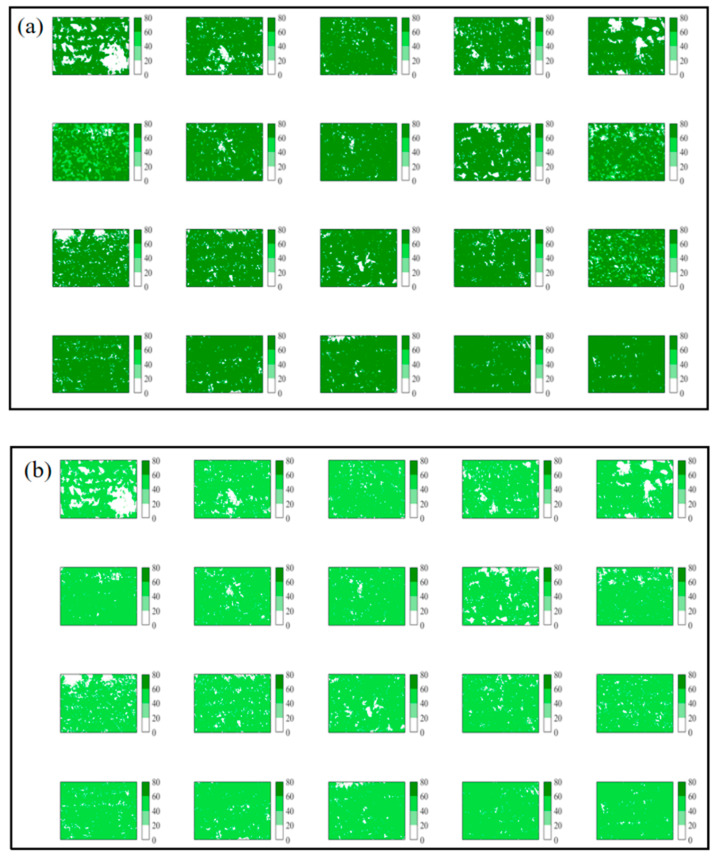

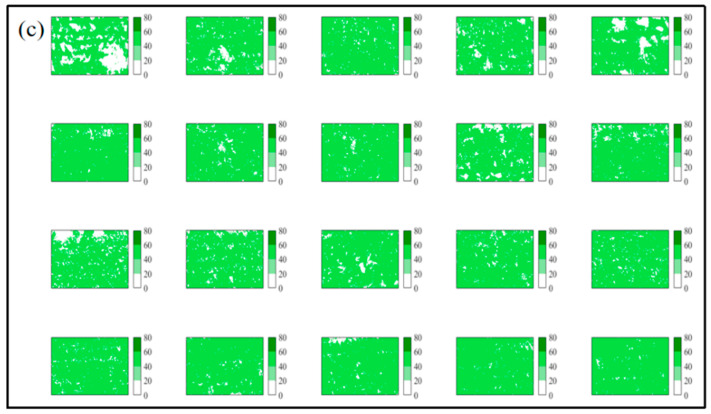
The predicted chlorophyll contents values using BP, SVM, and RF models using images acquired on 1 September 2019. (**a**–**c**) represented the predicted chlorophyll contents using BP, SVM, and RF, respectively.

Since the ML models can be perfectly applied and evaluated, thus the models were rebuilt using all samples for BP, SVM, and RF, respectively. All samples were used to build models and to predict the chlorophyll contents using all the VI calculated from RGB images acquired on 16 September 2019 (Figure 4). The results of predicted chlorophyll contents using BP, SVM, and RF for 8 July, 18 August, 1 September are shown in Figure A4, Figure A5 and Figure A6, respectively.

## 4. Discussion

### 4.1. Limitations in Assessing the Sscale Impacts

In this study, three approaches were applied to assess the scale effects from different flight altitudes with consideration of the disturbance of background (soil and grass) and color space system. The result was in accordance with previous studies that the second approach showed more regularity, and the precision had improved significantly as the applied method has been successfully conducted for classifying and extracting the green pixels [52,82]. However, the introduced approach can hardly completely exclude the interference of background, in other words, the extracted green pixels were not pure enough to have only including the green vegetation. Thus, the uncertainty from the background of soil remained and may have influenced the reliability of results to some extent. Meanwhile, the disturbance of grass can hardly be excluded as they were green pixels in pictures. The bidirectional reflectance distribution function (BRDF) has also been used for modeling light trapping in solar cells and it is commonly used for corrections of different angles of solar radiation. BRDF effect was commonly assessed in quantitative remote sensing. Since all images acquired in this study were almost strictly vertical to the ground and the imaging conditions were the same for image acquisitions. Thus, the BRDF was not investigated or assessed in this study and the impacts from BRDF can be ignored. We have focused on the assessments and evaluations of scale impacts of different resolutions ascribed from different flight altitudes [87,88]. Without considering the BRDF, the RGB images from flight altitude of 50 m will better fit the ground samplings. 

The lighting conditions were crucial factors influencing the quantitative remote sensing. In this study, the impacts from lighting conditions were limited as we have conducted two main processes before the assessment of scale impacts. First, the weather condition for acquiring images from the different flight altitudes were the same, thus, the lighting conditions during image acquisition was the same as we controlled the total flight time within 30 min. Thus, the only difference was the flight altitude, which was what we want to assess and evaluate. Secondly, we have converted the original DN values into normalized RGB values to eliminate the impacts of different lighting conditions. The scale impacts from different flight altitudes were of geometry, and the influence from different lighting conditions were limited. Therefore, we have enough reason to believe that the disturbance of lighting conditions for image acquisition can be eliminated to the least. However, the slight impacts of the lighting conditions will remain even though we have strictly controlled the total time of flight duration and the RGB values were normalized. However, the situations were different for assessing the estimations of chlorophyll contents using combined multi-vegetation index (VI) and ML. The lighting conditions for data acquisition were crucial for acquiring images of different days. The weather should be sunny, which is the basic requirement for both satellite and UAV quantitative remote sensing. If the weather conditions were different for different days of data acquisition, the impacts will be obvious. Thus, we suggest that the weather is better for assessing scale impacts and it should be sunny for acquiring the data for assessing the growth condition of maize.

The detailed parameters of the DJI UAV platform were introduced in Section 2.2.1. The spatial resolutions were 0.006, 0.018, 0.021, 0.028, 0.034 m for 25, 50, 75, 100 and 125 m, respectively. Since the flight altitudes of 50 m of this specific DJI platform were least influenced by the scale impacts, which meant that the spatial resolution of 0.018 m can precisely match the ground sampling of chlorophyll contents measured using SPAD-502. Thus, it is highly recommended that the spatial resolution should be optimized as 0.018 m to reveal the chlorophyll contents with the combined ground measurement using SPAD-502. Since the resolution of this camera mounted on DJI platform were 5472 × 3648 pixels, the flight altitudes should be higher than 50 m for camera with higher resolutions and the flight altitudes should be lower for camera with lower resolutions. The findings of this study may be helpful in future related agricultural and ecological studies such as monitoring the growth and predicting the yield of maize.

### 4.2. Machine-Learning-Based Chlorophyll Content Estimation

ML methods were widely used for regression and classification in RS domains as it can precisely catch the dynamic changes of the relationships between variables and input-output mapping. In this study, the BP, SVM, and RF were used for chlorophyll contents predictions using the VI calculated from RGB images acquired from flight altitude of 50 m that were least influenced by scale impacts. With 70% of ground samples for modeling and the remaining 30% of the sample for validating, the ML performed perfectly well and most of the predicted chlorophyll contents were within the expected ±15 error lines. However, the errors of some predicted values were relatively large using BP and the results had reached out of the error lines, which indicated that the errors of predicted values were relatively large. The SVM and RF models were better than the BP model, and this was due to the BP algorithm had disadvantages in balancing the prediction ability as it used a gradient steepest descent method, which may converge to local minimum [89,90]. Also, the BP may have the over-learning issue that may result in the “overfitting” problem [91,92]. The advanced SVM and RF models can balance the errors, obtaining robust and reliable results [93,94]. Thus, the SVM and RF models are suggested in application of agricultural yield predictions. 

With the development of more advanced ML techniques such as deep learning (DL), the solutions of regression and classification can be efficiently solved. The DL method can build more layers of complex fully connected deep models in predicting regressions. Among various DL methods, the convolutional neural network (CNN) is among the most common used in image processing. Thus, DL variants should be considered for agricultural and ecological applications (regression and predictions).

## 5. Conclusions

In this study, the scale impacts were first assessed using UAV RGB images acquired from five different flight altitudes and chlorophyll contents measured by SPAD-502. Three approaches were proposed by considering the effects of background and impacts of color space system, then the linear regression between the VI and chlorophyll contents of each plot were conducted. We found that the scale impacts of images acquired at the flight altitude of 50 m (spatial resolution 0.018 m) using DJI UAV platform with this specific camera (5472 × 3648 pixels) were least. Also, the HSV performed better than the traditional RGB and it can be used for information extraction. Three commonly used ML methods were adopted to conduct the pixel-based chlorophyll contents prediction at different growth stages of maize, and the SVM and RF performed better than the BP. We had provided a complete solution for predicting chlorophyll contents using combined UAV-RS and ML, and it is highly recommended that the integration of ML technology (SVM and RF) and UAV-based RGB images (acquired from 50 m for this DJI platform) should be adopted and applied for chlorophyll contents predictions in agricultural and ecological applications.

## Figures and Tables

**Table 1:** The VI evaluated in this study. R, G, B indicated normalized red, green, and blue bands, respectively. Note: α was a constant as 0.667.

Index	Name	Equation	Reference
E1	EXG	2×G−R−B	[50,51]
E2	EXR	1.4×R−G	[52]
E3	VDVI	(2×G−R−B)/(2×G+R+B)	[53,54,55,56]
E4	EXGR	2×G−R−B−1.4×R−G	[57]
E5	NGRDI	(G−R)/(G+R)	[55,58]
E6	NGBDI	(G−B)/(G+B)	[54,59]
E7	CIVE	0.441×R−0.8818G+0.385×B+18.787	[60,61]
E8	CRRI	G/R	[62,63,64]
E9	VEG	$G/R^{\alpha }B^{1-\alpha }$	[65,66]
E10	COM	0.25EXG+0.3EXGR+0.33CIVE+0.12VEG	[67,68]
E11	RGRI	G/B	[69,70]
E12	VARI	(G−R)/(G+R−B)	[71,72]
E13	EXB	1.4×B−G	[67,73]
E14	MGRVI	(G×G−R×R)/(G×G+R×R)	[74,75]
E15	WI	(G−B)/(R−G)	[72,76]
E16	IKAW	(R−B)/(R+B)	[58,77]
E17	GBDI	G − B	[52,78]
E18	RGBVI	(G × G − B × R)/(G × G + B × R)	[79,80,81]

**Table 2:** The calculated R^2^ between VI and chlorophyll contents using images acquired from different dates. Note: the data represents the time when the used images were acquired and E1–E18 are in corresponding with the VI in Table 1.

**Date**	**E1**	**E2**	**E3**	**E4**	**E5**	**E6**	**E7**	**E8**	**E9**
**8 July**	0.182	0.139	0.181	0.169	0.185	0.177	0.178	0.181	0.178
**18 August**	0.240	0.499	0.210	0.091	0.514	0.362	0.040	0.506	0.530
**1 September**	0.273	0.648	0.228	0.487	0.629	0.291	0.001	0.581	0.606
**16 September**	0.471	0.832	0.462	0.722	0.845	0.342	0.047	0.825	0.842
**Date**	**E10**	**E11**	**E12**	**E13**	**E14**	**E15**	**E16**	**E17**	**E18**
**8 July**	0.179	0.181	0.186	0.170	0.185	0.170	0.202	0.182	0.182
**18 August**	0.010	0.506	0.733	0.365	0.493	0.729	0.804	0.001	0.211
**1 September**	0.003	0.581	0.671	0.400	0.622	0.591	0.674	0.263	0.314
**16 September**	0.103	0.825	0.751	0.450	0.849	0.855	0.838	0.481	0.534

**Table 3:** The detailed results of R^2^, RMSE, and MAE for different dates using ML methods.

**R^2^**	**8 July**	**18 August**	**1 September**	**16 September**
BP	0.001	0.454	0.595	0.703
SVM	0.001	0.332	0.587	0.702
RF	0.001	0.227	0.465	0.599
**RMSE**	**8 July**	**18 August**	**1 September**	**16 September**
BP	3.868	3.533	3.411	4.600
SVM	2.500	3.575	3.328	3.043
RF	2.622	2.541	3.333	3.095
**MAE**	**8 July**	**18 August**	**1 September**	**16 September**
BP	2.973	2.765	2.347	3.701
SVM	1.802	2.757	2.844	2.438
RF	2.174	2.138	2.736	2.509

## Appendix A

**Table A1:** The spatial distribution of fertilizer at 20 plots during the growth of maize. The NPK meant different combinations of fertilizers, and N represented the nitrogenous fertilizer, P represented the phosphate fertilizer, K represented the potassium fertilizer, respectively. The number after NPK represents the actual multiple of fertilizer fertilizers, and the numbers in brackets were the number of plots in corresponding with the plots shown in Figure A1.

	Column 1	Column 2	Column 3	Column 4
**Row 1**	N2+straw (4)	N3P3K1 (3)	N3P1K1 (2)	N1P1K2 (1)
**Row 2**	N2+Organic fertilizer (8)	N3P2K1 (7)	N3P3K2 (6)	N1P1K1 (5)
**Row 3**	N3+straw (12)	N4P3K1 (11)	N2P2K2 (10)	N1P2K1 (9)
**Row 4**	N3+Organic fertilizer (16)	N4P2K1 (15)	N2P1K1 (14)	N1P3K1 (13)
**Row 5**	N4P2K2 (20)	N4P1K1 (19)	N2P2K1 (18)	N2P3K1 (17)

**Table A2:** The R^2^ between vegetation indices calculated from RGB and chlorophyll contents using the images with background.

RGB Index with Background	25 m	50 m	75 m	100 m	125 m
**index1**	0.146	0.154	0.133	0.114	0.096
**index2**	0.100	0.119	0.121	0.096	0.092
**index3**	0.148	0.171	0.156	0.144	0.117
**index4**	0.115	0.130	0.125	0.102	0.094
**index5**	0.109	0.129	0.133	0.109	0.093
**index6**	0.203	0.173	0.159	0.166	0.126
**index7**	0.143	0.152	0.132	0.113	0.096
**index8**	0.105	0.141	0.134	0.130	0.104
**index9**	0.102	0.130	0.143	0.122	0.115
**index10**	0.124	0.137	0.127	0.106	0.095
**index11**	0.105	0.141	0.134	0.130	0.104
**index12**	0.105	0.126	0.131	0.106	0.091
**index13**	0.214	0.199	0.149	0.137	0.098
**index14**	0.102	0.127	0.137	0.121	0.096
**index15**	0.057	0.057	0.002	0.024	0.079
**index16**	0.007	0.002	0.081	0.067	0.048
**index17**	0.148	0.171	0.156	0.144	0.117
**index18**	0.147	0.153	0.132	0.114	0.096

**Table A3:** The R^2^ between vegetation indices calculated from RGB and chlorophyll contents using the images without background.

RGB Index without Background	25 m	50 m	75 m	100 m	125 m
**index1**	0.172	0.181	0.160	0.142	0.119
**index2**	0.008	0.058	0.046	0.059	0.042
**index3**	0.180	0.189	0.162	0.144	0.118
**index4**	0.188	0.206	0.162	0.141	0.116
**index5**	0.187	0.181	0.153	0.142	0.118
**index6**	0.153	0.176	0.166	0.141	0.119
**index7**	0.157	0.156	0.129	0.122	0.099
**index8**	0.166	0.163	0.136	0.127	0.104
**index9**	0.165	0.162	0.134	0.124	0.102
**index10**	0.157	0.157	0.130	0.122	0.099
**index11**	0.166	0.163	0.136	0.127	0.104
**index12**	0.192	0.181	0.150	0.142	0.117
**index13**	0.123	0.131	0.062	0.084	0.075
**index14**	0.187	0.181	0.153	0.142	0.118
**index15**	0.120	0.157	0.153	0.129	0.115
**index16**	0.151	0.179	0.087	0.127	0.107
**index17**	0.172	0.180	0.159	0.142	0.119
**index18**	0.181	0.189	0.162	0.144	0.118

**Table A4:** The R^2^ between vegetation indices calculated from HSV and chlorophyll contents using the images without background.

HSV Index without Background	25 m	50 m	75 m	100 m	125 m
**index1**	0.149	0.179	0.166	0.155	0.159
**index2**	0.151	0.179	0.167	0.154	0.163
**index3**	0.151	0.179	0.167	0.154	0.163
**index4**	0.151	0.179	0.167	0.154	0.163
**index5**	0.117	0.174	0.157	0.146	0.166
**index6**	0.109	0.170	0.153	0.140	0.163
**index7**	0.145	0.175	0.164	0.150	0.158
**index8**	0.145	0.175	0.164	0.150	0.158
**index9**	0.150	0.177	0.166	0.151	0.162
**index10**	0.150	0.177	0.166	0.151	0.162
**index11**	0.150	0.177	0.166	0.151	0.162
**index12**	0.150	0.177	0.166	0.151	0.162
**index13**	0.150	0.177	0.166	0.151	0.162
**index14**	0.150	0.177	0.166	0.151	0.162
**index15**	0.149	0.174	0.165	0.148	0.162
**index16**	0.109	0.170	0.153	0.140	0.163
**index17**	0.064	0.164	0.137	0.126	0.163
**index18**	0.149	0.174	0.165	0.148	0.162
